# Mapping the extent and nature of research examining health literacy and ethnic inequalities in maternity care: a scoping review

**DOI:** 10.1186/s12913-026-15233-3

**Published:** 2026-08-25

**Authors:** B. McEvoy, K. Watson, E. Aalai, G. Rowlands, H. Soltani, A.E.P. Heazell

**Affiliations:** 1 Saint Mary’s Hospital, Manchester University NHS Foundation Trust, Oxford Road, Manchester, M13 9WL UK; 2 Division of Nursing, Midwifery and Social Work, School of Health Sciences, Faculty of Biology, Medicine and Health, University of Manchester, Manchester, UK; 3 Public Health Sciences Institute, Faculty of Medical Sciences, Newcastle University, Newcastle upon Tyne, UK; 4 Department of Nursing and Midwifery, Sheffield Hallam University, Sheffield, UK; 5 Maternal and Fetal Health Research Centre, Division of Developmental Biology and Medicine, School of Medical Sciences, Faculty of Biology, Medicine and Health, University of Manchester, Oxford Road, Manchester, M13 9WL UK

**Keywords:** Ethnic inequalities, Maternity care, Midwifery, Obstetrics, Health literacy

## Abstract

**Background:**

Globally, women from ethnic minority groups experience stark disparities in maternity care outcomes. While ethnic inequalities are complex, health literacy is a contributing factor. This scoping review aimed to map the extent and nature of research examining health literacy and ethnic inequalities in maternity care. The review sought to identify evidence gaps and inform the direction of future research in this area.

**Methods:**

This scoping review followed methodological guidance published by Arksey and O’Malley (2005). A comprehensive search across 5 key databases and relevant grey literature identified 1,024 citations. Papers were reviewed against criteria for inclusion. Key study features were collated in a standardised data extraction form. Thematic analysis summarised key findings across the studies.

**Results:**

The review included 14 papers that directly explored health literacy and ethnic inequalities in maternity care. The research had a wide geographical distribution and included quantitative (*n* = 7), qualitative (*n* = 4) and mixed methods research designs (*n* = 3). Data were collected using a range of health literacy assessment tools. The focus of the research varied across the scope of maternity care, including pregnancy (*n* = 7), the postnatal period (*n* = 2) and the whole maternity continuum (*n* = 5). Thematic analysis summarised four key findings: (i) Diverse definitions of health literacy; (ii) Health literacy challenges can disproportionately affect minority ethnic women; (iii) Poor health literacy can contribute to adverse outcomes and ethnic inequalities and (iv) Limited evaluation of health literacy interventions.

**Conclusions:**

Health literacy is an important but understudied contributor to ethnic inequalities in maternity care. There is a need for high-quality in-depth research to explore health literacy and ethnic inequalities in maternity care. This will support the development of targeted, equity focused interventions. Optimisation of health literacy may serve to reduce persistent inequalities experienced by minority ethnic groups in maternity care outcomes.

## Statement on language

Throughout this paper the term ‘minority ethnic women’ will be used to describe women who are from an ethnic minority group, within the study setting. A detailed description of ethnicity will be prioritised, and collective terminology will only be used where this is not possible. The terms ‘women’ and ‘woman’ are used in this paper, which should be taken to include people who do not identify as women but are pregnant or have given birth.

## Background

Ethnic inequalities in maternity care outcomes and experiences represent a significant global health challenge. Rates of maternal mortality, stillbirth, neonatal death, preterm birth and other adverse outcomes are significantly higher for minority ethnic women across high- and low-income countries [1–3]. For example, in the United States, Black women have significantly more pregnancy related deaths when compared to White, Hispanic and Asian women [4].

Similarly, in the United Kingdom, maternal mortality rates are almost twice as high for Asian women, and three times higher for Black women when compared to White women [5]. Babies from Black ethnic backgrounds are also more than twice as likely to be stillborn (6.19 vs. 2.99 per 1,000 total births) [6]. These disparities extend to the prevalence of postpartum haemorrhage, mental health complications, preterm birth, and birth trauma [5–8].

The causes of ethnic inequalities are complex and are impacted by systemic racism [9], socioeconomic disadvantage, burden of comorbidities, suboptimal and discriminatory care [10–15]. Although ethnicity is not synonymous with deprivation or culture, the intersection of these factors can compound disadvantage and create multiple barriers to care [11–15]. Understanding these intersecting factors is crucial for developing targeted interventions to reduce inequalities.

Health literacy has been identified as a contributing factor to inequalities. Health literacy describes a person’s capacities to access, understand and use health services and information to make informed decisions about their health. It encompasses personal attributes such as knowledge, understanding, social support, communication, appraisal and advocacy skills [16, 17]. It also includes personal experiences and organisational factors that can impact how people navigate health systems and services [16, 17]. Such organisational factors include: complicated care pathways, culturally inappropriate resources, and ineffective communication between healthcare professionals and service users.

Lower health literacy is associated with lower life expectancy, increased hospital admissions, lower levels of self-rated health, multimorbidity, higher levels of chronic illness and for those living with chronic illness, a greater impact on everyday life [18]. Statistical modelling suggests that improving health literacy could mitigate some of these effects and contribute to tackling disparities in health outcomes [19]. Consequently, the World Health Organisation recognises health literacy as a modifiable determinant of health. Health systems are often not responsive to the needs of diverse populations, and access and use of services may need a degree of literacy greater than some service users possess. Moreover, health literacy challenges are experienced unequally, with people from minority ethnic groups, those from lower socioeconomic backgrounds and those with limited language proficiency experiencing higher rates of health literacy challenges [20, 21].

Within the context of maternity care, lower health literacy has been associated with ethnic inequalities [21, 22] including increased risk of stillbirth, maternal and neonatal death, after controlling for socioeconomic factors [23]. There is call for increased research into health literacy and maternity care, with a focus on developing evidence-based interventions to optimise health literacy for ethnic minority populations [21–25]. To address this call, a scoping review was undertaken to map the extent and nature of research examining health literacy and ethnic inequalities in maternity care.

This scoping review differs from existing reviews in health literacy by being sited within the context of ‘maternity care’; the population of focus is women receiving maternity care. Previous systematic reviews have explored health literacy in the general pregnant population, without a focus on ethnic inequalities [21, 24, 25]. Other reviews have aimed to assess level and impact of health literacy on minoritised pregnant women with narrow focus on antenatal care [22], language proficiency [20] and the incidence of stillbirth and adverse outcomes [25]. Whilst these reviews offer insight into this topic area, many of the primary research studies did not explore health literacy and ethnic inequalities across the whole continuum of maternity care, regardless of a focused topic or area of maternity care. Thus, this scoping review addresses a distinct gap by exploring health literacy as a defined concept and its intersection with ethnic inequalities across the full maternity care continuum. The overarching purpose of this review was to identify evidence gaps and inform the direction of future research aiming to address ethnic inequalities in maternity care.

## Aim

To map the extent and nature of research examining health literacy and ethnic inequalities in maternity care.

## Methods

This scoping review followed methodological guidance published by Arksey and O’Malley [26] which aims to map the scope and nature of research underpinning a topic area. The scoping review adheres to the Preferred Reporting Items for Systematic Reviews and Meta-Analyses extension for Scoping Reviews (PRISMA-ScR) guidelines [27] to ensure transparent and comprehensive reporting. A protocol for the review was registered retrospectively (https://osf.io/95wyz/overview) because the methodology followed an exploratory approach and required flexibility to map the scope of evidence in this field.

### Literature search

A comprehensive search strategy was developed and guided by the Population Concept Context (PCC) framework [28] (Table 1). The population of interest was ‘minority ethnic women’, the concept was ‘health literacy’, and context was ‘maternity care’. Key search terms were identified through preliminary exploration of the literature and in consultation with a medical academic librarian. Medical Subject Headings (MeSH) terms and free-text terms were combined using Boolean operators.

**Table 1:** Search strategy and terms used to search databases

**Population**	ethnic minority wom? n* OR Asian wom? n* OR Black wom? n* OR BME OR BAME OR migrant OR ethnic* OR multi-ethnic OR multi-cultur* OR raci*
**Concept**	Health literacy or medical literacy
**Context**	Matern* or perinatal or prenatal or antenatal or intranatal or postnatal or obstetric* or pregnan* or childbear* or childbirth or antepartum or intrapartum or postpartum or? partum or midwif* or deliver* or labo? r or parturition

The search was conducted across 5 key databases (Cinahl, Embase, Maternity and Infant care, LILACS and Medline). Grey literature searches were conducted through the university library service, Google Scholar, and relevant websites to ensure completeness. A final search was completed in September 2025. Additional citation searching examined reference lists of included studies and relevant literature [20–25].

### Eligibility and selection

Papers were screened for eligibility by the primary author following an inclusion and exclusion criteria (Table 2). Inclusion was limited to primary research studies exploring health literacy in maternity care contexts (encompassing pregnancy, birth, or postnatal periods) and with a focus on ethnic inequalities. Ethnic inequalities were defined as disparities experienced by ethnic minority groups within the study setting.

**Table 2:** Inclusion and exclusion criteria for the scoping review

Inclusion criteria	Exclusion criteria
• Primary research • Research directly exploring ‘health literacy’ • Research focused on maternity care contexts (pregnancy, birth and/or postnatal period) • Research exploring ‘ethnic inequalities’ defined as disparities experienced by ethnic minority groups within the study setting • Research published in the last 10 years	• Secondary research • Research that does not directly explore ‘health literacy’ • Research that falls outside of maternity care contexts (e.g.: pre-conception) • Research published before 2015

Narrative literature reviews, commentaries and opinion-based studies were excluded, though the list of references in reviews were screened to ensure inclusion of all relevant studies. Search limits were applied to include studies published in English, involving human participants and focusing on female populations. A date restriction was applied to include studies published from 2015 through to September 2025. This was a pragmatic decision selected to capture research that reflects contemporary understandings of health literacy being a multifaceted concept, as described in the World Health Organization’s 2015 ‘Health Literacy Toolkit’ [29]. This encompasses personal skills, characteristics, wider resources, organisational factors and support required to effectively manage health [29].

### Data extraction and analysis

A standardised data extraction tool was developed to capture the study aim, research design, setting, population and maternity focus, in addition to key findings relating to health literacy and ethnic inequalities in maternity care. The purpose of this scoping review was to map the breadth of the literature rather than to produce a formal quality judgement or exclude studies based on methodological assessment. Although critical appraisal using a formal classification system was not undertaken, the primary author documented the methodological strengths and limitations of individual studies within the data extraction tool to provide context for the findings and identify evidence gaps. This included definitions of health literacy and measurement tools used to assess health literacy.

Arksey and O’Malley [26] recommend thematic summarisation of findings to highlight key themes, but not to synthesise evidence in the same way as a systematic review. In this review thematic analysis included data familiarisation, where full papers were read to understand the breadth and scope of the evidence, before recurring concepts and findings were organised into themes summarising the nature and extent of the knowledge in the topic area. While methodological observations informed the interpretation of evidence, they were not used to assess quality or prioritise studies, limiting the scope of this review to a mapping exercise rather than an evidence synthesis.

## Results

**Fig. 1:**
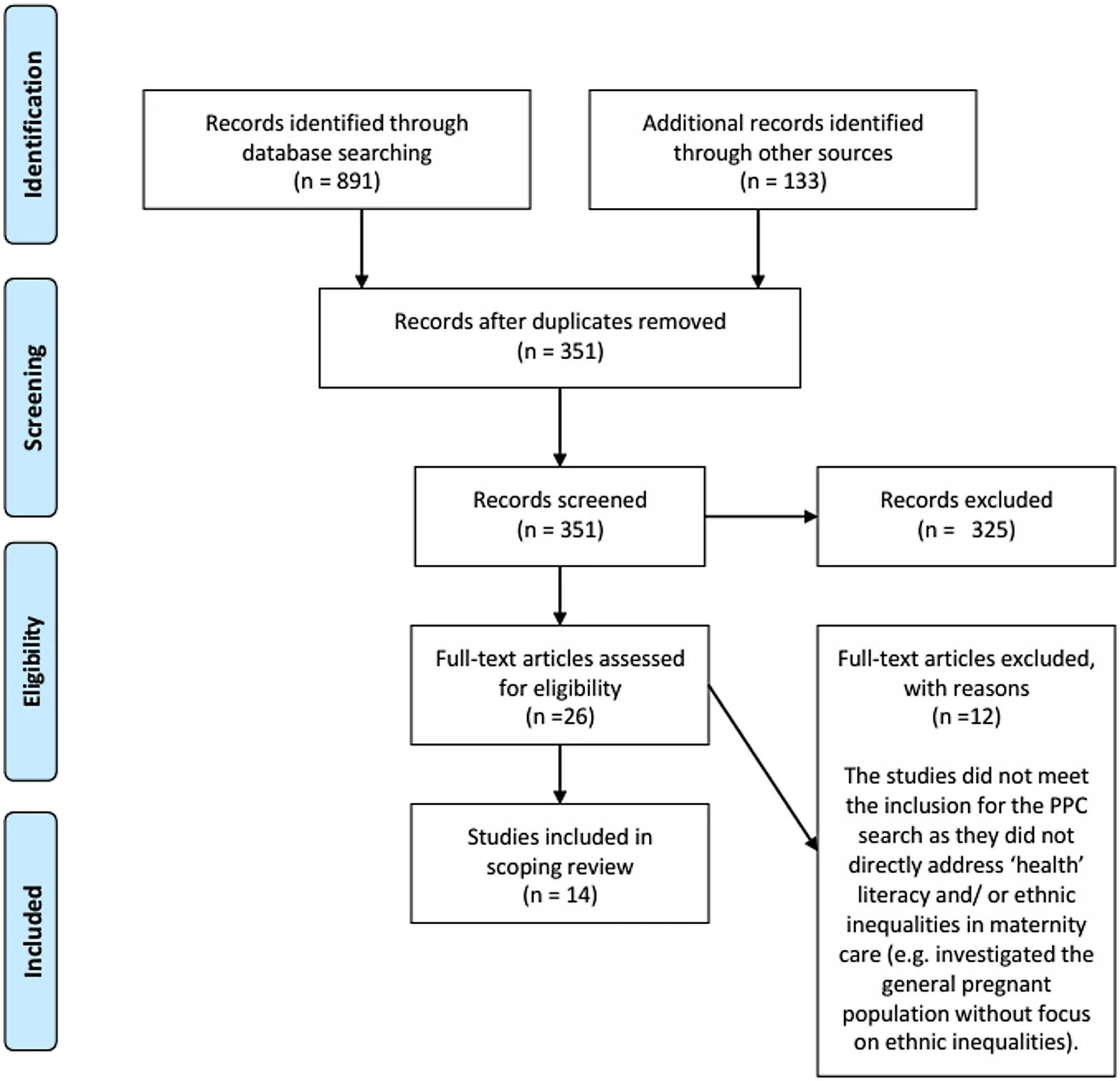
PRISMA flow diagram detailing the study selection process

In total the searches found 1,024 citations. After duplicate removal and screening of titles and abstracts, 26 papers were assessed in full, with 14 meeting the criteria for inclusion. Titles, abstracts and full papers were screened independently by the primary author (BM) with any uncertainties resolved through discussion with the wider research team. The main reason for exclusion of papers was that they did not directly explore ‘health literacy’ as a concept or did not focus on ethnic inequalities. Figure 1 presents the PRISMA flow diagram detailing the selection process.

### Study characteristics

**Fig. 2:**
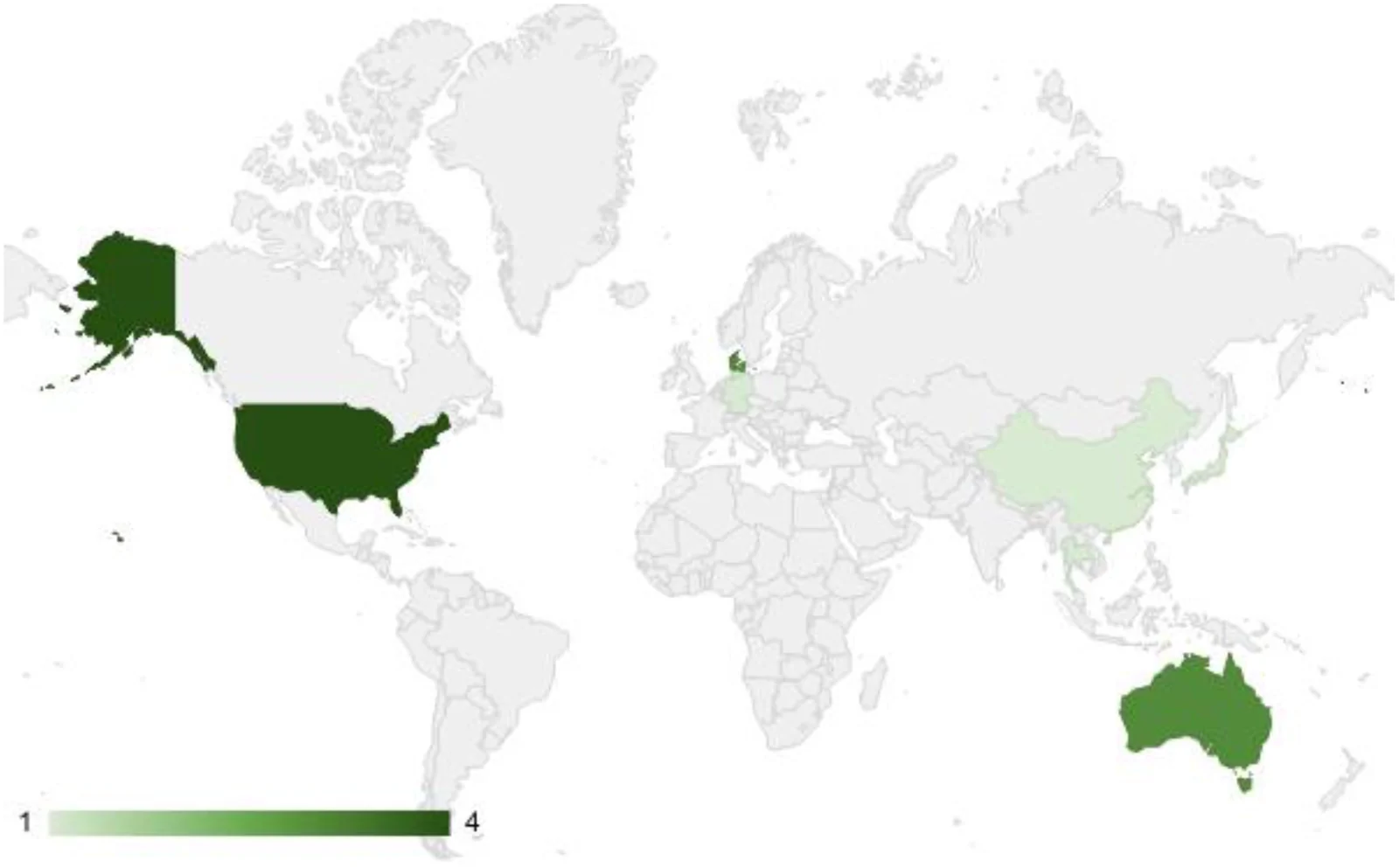
Illustrates the geographical distribution of research examining health literacy and ethnic inequalities in maternity care

The study characteristics, including methodological strengths and limitations are summarised in Table 3. The 14 research studies were conducted in Denmark (*n* = 3) [30–32], Australia (*n* = 3) [33–35], United States (*n* = 4) [36–39], Germany (*n* = 1) [40], Japan (*n* = 1) [41], Thailand (*n* = 1) [42] and China (*n* = 1) [43] (Fig. 2).

**Table 3:** Standardised data extraction table

Aim	Design and health literacy measurement tool	Setting	Ethnic minority population focus	Maternity focus	Key Findings	Strengths/Limitations	Health literacy definition (and measurement tools)
To explore experiences of non-Western ethnic minority pregnant women with Gestational Diabetes and information integration **(Dayyani et al.**,** 2019)**	Qualitative | Semi-structured interviews (*n* = 11)	Denmark	Non-Western pregnant women with Gestational Diabetes	Pregnancy (topic of Gestational Diabetes)	Three themes: reaction to diagnosis, everyday life, information needs. Women felt sad, worried and struggled with behaviour changes, some lacked spousal support. Hospital information was positively received but sometimes misunderstood. Low HL and/or poor Danish language skills made the women more challenged by diagnosis.	Small sample; rich qualitative insights into lived experience of minority ethnic women; use of qualitative methodology phenomenology; focus on topic of gestational diabetes limiting application to wider inequalities in maternity care.	Defines health literacy as multifaceted including HL as functional (read and use numbers), interactive (discuss and interact) and critical (capacity to act) in addition to contribution of others to support |Qualitative assessment of health literacy based on questions drawn from the HLS-EU-Q47 questionnaire and women’s description of their experience.
To identify health literacy issues in maternity care for culturally and linguistically diverse women and needed strategies **(Hughson et al.**,** 2018)**	Qualitative | Case study with semi-structured interviews with healthcare professionals (*n* = 18 clinicians)	Australia	Culturally and linguistically diverse mothers (healthcare professionals interviewed)	Holistic maternity care (pregnancy to postnatal)	Three barriers patient factors (communication, cultural barriers, access), provider factors (time constraints, interpreter issues), enablers (cultural awareness, technology)	Study formed part of a wider needs assessment but did employ a health literacy framework for analysis. Healthcare provider perspective only; systematic issues identified; an exploratory qualitative approach but no specific qualitative methodology applied.	Defines health literacy as individual’s health literacy (skills, knowledge, motivation, access, appraise, make decisions about health care) and the health literacy environment (policies, processes, people in the health system that impact health literacy) | No health literacy assessment tool but framework used for analysis.
To explore health literacy in maternity care for forced migrant women **(Mohr et al.**,** 2023)**	Qualitative | Secondary analysis of healthcare professionals interviews (*n* = 59)	Germany (Berlin, Brandenburg, North Rhine Westphalia)	Forced migrant women (healthcare professionals interviewed)	Holistic maternity care (pregnancy to postnatal)	Barriers present at healthcare and personal levels. Health literate care needs to be culturally sensitive and include migrant women.	Secondary analysis of qualitative data limits depth of exploration into health literacy; comprehensive sample size; healthcare professional perspective only.	Defines health literacy as “people’s knowledge, motivation and competences to access, understand, appraise, and apply health information”) | No health literacy assessment tool but framework used for analysis.
To co-design multi-language maternal health education videos for refugee/migrant women **(Bartlett et al.**,** 2022)**	Qualitative | Design thinking methodology in 3 stages using focus groups (*n* = 26 participants)	Australia, Melbourne	Refugee and migrant women (mix of 16 healthcare staff, 8 Arabic/Dari-speaking women, 2 bicultural educators took part in focus groups)	Holistic maternity care (pregnancy to postnatal)	Produced 5 posters, 4 videos, 4 recommendations. Demonstrated collaborative design can address health literacy gaps.	Limited methodological detail; valuable co-design approach produced collaborative health literacy resources; mix of health care professionals and women’s perspectives.	The study does not offer a detailed definition of health literacy or discuss elements of health literacy that were evaluated. Based on the aims, it appears health literacy was a term used to describe knowledge, navigating, and understanding.
To assess mental health literacy of perinatal Hispanic adolescents using modified mental health literacy scale **(Recto et al.**,** 2017)**	Quantitative | Cross-sectional descriptive study (*n* = 30 participants) surveyed using a Modified Mental Health Literacy scale and demographic questionnaire	United States, San Antonio	Hispanic pregnant/postpartum adolescents (14–21 years) in the United States	Holistic maternity care (pregnancy to postnatal)	High rates of self-reported depression during perinatal period; no significant sociodemographic differences, those with depression had higher mental health literacy scores.	Small convenience sample; modified tool used which may impact validity; focused sample on pregnant and postpartum adolescents which may impact generalisability.	Defines mental health literacy to include (a) recognition of disorders, (b) knowledge of risk factors, (c) knowledge and beliefs of self-help strategies, (d) knowledge of professional help/ treatment (e) attitudes promoting recognition and help-seeking, (f) knowledge of how to seek information | Modified Mental Health Literacy Scale questionnaire.
To identify social media use patterns and explore health/eHealth literacy influence **(George et al.**,** 2023)**	Quantitative | Cross-sectional survey (*n* = 404 participants) including Single Item Literacy Screener and ‘eHEALS’ scale to measure eHealth literacy	United States	Black pregnant and postnatal women (*n* = 404) in the United States	Pregnancy (topic of social media use to obtain information about pregnancy)	67.5% had high health literacy; women with low health literacy used social media more for pregnancy information and support; high eHealth Literacy associated with information sharing with professionals.	Large representative sample; validated tools used; validated measure but can be considered a limited measure of health literacy because it only asks about one aspect of health literacy through one question; risk of recall bias for women who were up to a year postnatal.	Defines Health literacy as ability to “obtain, process, understand and communicate about health-related information needed to make informed decisions” | Item Literacy Screener (SILS) and eHEALS scale was used to measure eHealth literacy.
To examine gestational weight gain health literacy in immigrant vs. native Japanese mothers **(Kigawa et al.**,** 2024)**	Quantitative | Longitudinal cohort study, matched analysis, using a self-administered questionnaire (*n* = 67,953 women)	Japan	Migrant pregnant women (324 immigrant, 963 native matched mothers)	Pregnancy (topic of Gestational Weight Gain)	Migrant mothers had less gestational weight gain knowledge but appropriate weight gain and fewer low birth weight infants, suggesting other protective factors.	Large sample; baseline characteristics similar in both groups; knowledge-based HL assessment may be limited; minority groups ‘non-native’ lacking detailed description of ethnicity.	Defines health literacy based on knowledge of gestational weight gain, rather than broader definition | Knowledge based questionnaire used rather than formal assessment of health literacy.
To evaluate MAMAACT intervention effect on health literacy and complication management **(Rasmussen et al.**,** 2023)**	Quantitative | Cluster randomised control trial of an intervention, Cross-sectional survey data were collected using telephone interviews, including 2 Health Literacy Questionnaire domains (active engagement and navigating the system) (*n* = 4,150 women)	Denmark (19/20 maternity wards)	Migrant pregnant women (670 non-Western immigrant)	Pregnancy (topic of pregnancy complications)	Intervention improved certainty about complication response but not health literacy levels of ‘active engagement’ or ‘navigating healthcare system’.	Robust randomised control trial design; validated Health Literacy Questionnaire; targeted recruitment of immigrant women	Defines health literacy as an individual’s ability to access, understand and use health-related information to make decisions about health | Validated Health Literacy Questionnaire.
To examine ethnicity association with active engagement with healthcare providers **(Brorsen et al.**,** 2022)**	Quantitative | Cross-sectional survey using the Health Literacy Questionnaire domain active engagement (1898 women)	Denmark (19/20 maternity wards)	Pregnant Danish, European, African, Asian women in Denmark (key variable of interest was maternal ethnicity)	Pregnancy (topic of engaging with care)	Immigrant women reported lower active engagement than ethnic Danish women	Large multilingual sample from maternity wards across Denmark; use of recruitment videos in 6 languages supported diverse recruitment; some small ethnic group sizes; only measured one domain of health literacy.	Defines health literacy as the combination of personal competencies and situational resources needed for people to access, understand, appraise and use information and services to make decisions about health | Validated Health Literacy Questionnaire.
To assess association between maternal health literacy and maternal/neonatal outcomes **(Yee et al.**,** 2021)**	Quantitative | Secondary analysis of multicentre cohort study using a Rapid Estimate of Adult Literacy in Medicine-Short Form test (*n* = 10038 women)	United States	Nulliparous individuals from White, Black, Hispanic and Asian backgrounds (key variable of interest was maternal ethnicity)	Holistic maternity care (pregnancy to postnatal outcomes)	Inadequate health literacy associated with younger age, less education, public insurance, underrepresented ethnicity, and various adverse maternal/neonatal outcomes.	Large multicentre study; large sample representative of the US population; observational study does not account for confounding; data collected mid pregnancy and health literacy skills may develop.	Defines HL as health care access and use, patient-practitioner interactions, and health behaviour, each of which may be influenced by patient and health system factors | validated ‘REALM-SF’ tool Rapid Estimate of Adult Literacy in Medicine Short Form.
To determine if computer-based modules can increase preterm birth health literacy **(Vanderbilt et al.**,** 2015)**	Quantitative | Pre-test and post intervention pilot study using a 10-point survey test (*n* = 140 participants)	United States, Richmond Metro	140 participants from underserved communities (large proportion from minority ethnic backgrounds)	Pregnancy (topic of pre-term birth)	Participants demonstrated increased knowledge in health literacy and preterm birth.	Pilot study limited evidence relating to overall research question and focus on research design; participants were from one area in Virginia limiting generalisability.	The study does not define health literacy, but measures health literacy based on knowledge scores | Knowledge-based health literacy measure.
To identify maternal health literacy status and influencing factors **(Chen et al.**,** 2025)**	Mixed methods | Explanatory sequential, using a quantitative survey (*n* = 501 women) and qualitative interviews (*n* = 16 women)	China, Guangzhou urban villages	Postpartum women (focus on migrant women, compared to women who were ‘registered’ nationals)	Postnatal	Ethnicity, education, income, registration status, delivery mode, and social support influenced health literacy scores. Ethnic minorities had lower health literacy rates; critical health literacy needs improvement.	Mixed methods design supported understanding of quantitative findings; large sample.	Defines health literacy including functional, communicative and critical health literacy | Structured questionnaire based on Squiers L health literacy skills framework.
To implement and evaluate pilot health literacy intervention for newly arrived migrant mothers **(Dougherty et al.**,** 2021)**	Mixed methods | evaluation of culturally redesigned parent classes using questionnaire (including 3 Health Literacy Questionnaire domains) and focus groups (*n* = 30 participants)	Australia, Sydney	Bangla/Mandarin speaking postnatal mothers in Australia	Postnatal	Higher post-intervention health literacy scores: nurses reported increased awareness of access barriers; scalable potential.	Community connector recruitment; small sample for pilot study; Health literacy questionnaire was translated into Bangla but not validated for this population.	Defines dimensions of health literacy (specifically, knowledge and access to information), as well as situational determinants of health literacy (specifically, social support) | Validated Health Literacy Questionnaire.
To conduct comprehensive health literacy assessment using folic acid campaign case study **(Gilder et al.**,** 2019)**	Mixed method | health literacy assessment using reading and comprehension tools (*n* = 525 women) and focus groups (*n* = 42 women)	Thailand-Myanmar border	Pregnant women from mostly migrant and ethnic minority backgrounds	Pregnancy (topic of folic acid supplementation)	Low health literacy prevalent; significant communication barriers; common poster misunderstanding highlights need for piloting materials.	Triangulation of methods provided comprehensive understanding of focused topic area; marginalized population recruitment of large sample size.	Defines health literacy as a multifaceted concept, including personal characteristics, comprehension skills and organisational factors | Health literacy assessed through reading fluency and comprehension.

**Fig. 3:**
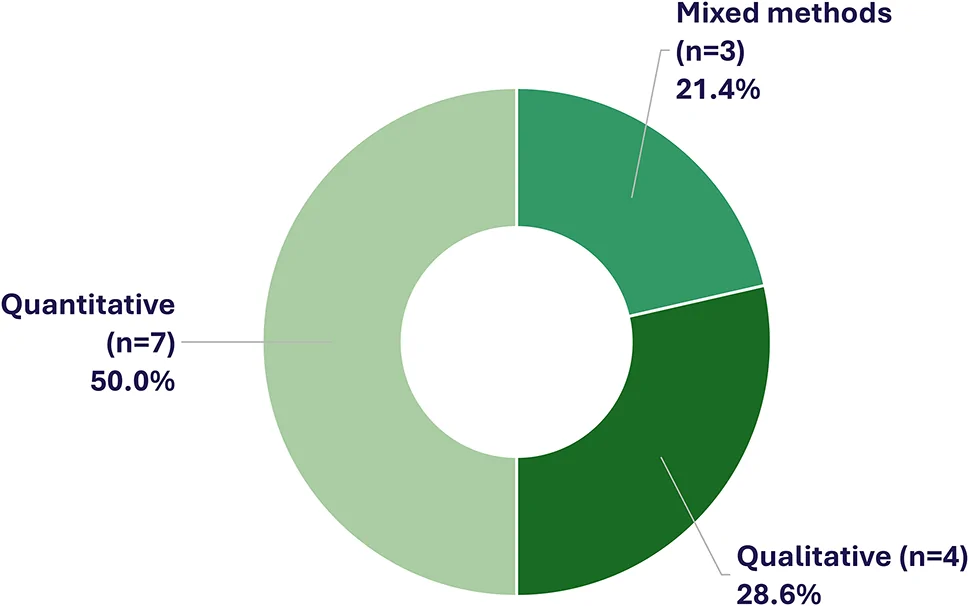
Range of research methodologies used to examine health literacy and ethnic inequalities in maternity care

**Fig. 4:**
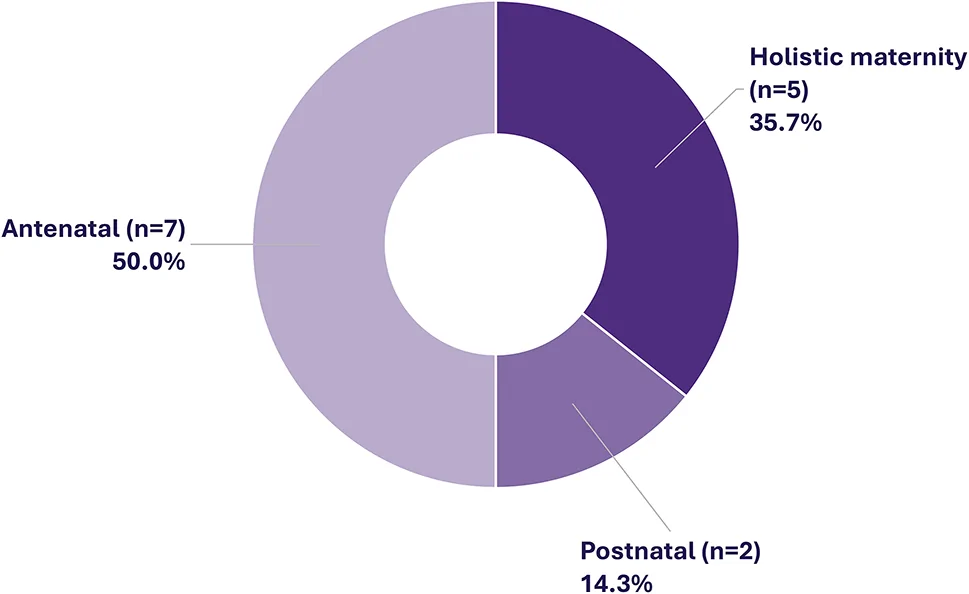
The focus of research across the maternity care continuum (antenatal, postnatal, whole continuum of maternity care)

The 14 papers included for review used a range of research methodologies (Fig. 3). Quantitative studies (*n* = 7) included a cluster randomized controlled trial [30], a cohort study [36, 41], survey, questionnaire [31, 37, 39] and descriptive research design [38]. Mixed-methods studies (*n* = 3) collected data through surveys [34, 42, 43], focus groups [34, 42] and interviews [43]. Qualitative research (*n* = 4) included a secondary analysis of interviews [40] and data collection through semi-structured interviews [32, 35] and focus groups [33]. Broadly, the studies aimed to explore health literacy levels and challenges among minoritised populations [31, 37, 38, 40, 42, 43], understand the impact of health literacy on care and health outcomes [32, 35, 36, 41], develop [33] and evaluate a health literacy intervention [30, 34, 39]. The focus of primary research varied across the maternity care continuum, from pregnancy through to the postnatal period (Fig. 4). Health literacy was explored across the whole maternity continuum in five studies [33, 35, 36, 38, 40]. Most of the literature focused on health literacy during pregnancy (*n* = 7) with a focus on topics including gestational diabetes [32], use of social media [37], gestational weight gain [41], pregnancy complications [30], engaging with maternity care [31], pre-term birth [39] and folic acid supplementation [42]. Only two studies explored health literacy in the postnatal period [34, 43]. No research specifically explored health literacy around the intrapartum period.

Data were collected from pregnant women [30–32, 36, 41, 42], postnatal women [34, 43] and health care professionals [33, 35, 40]. Two studies collected data from pregnant and postnatal women [37, 38]. One study collected data around a specific element of maternity care (pre-term birth) in underserved populations but did not specify if the population were antenatal or postnatal [39]. Established health literacy assessment tools were used in seven studies and included the Squiers Health Literacy Skills framework [43], the Rapid Estimate of Adult Literacy in Medicine-Short Form [36], a Single Item Literacy Screener (SILS) and eHEALS eHealth Literacy scale [37], a Modified Mental Health Literacy Scale [38] and a validated Health Literacy Questionnaire (HLQ) [30, 31, 34]. A qualitative study used the European Health Literacy Questionnaire (HLS-EU-Q47) as inspiration for qualitative questions [32]. Two studies [39, 41] used knowledge-based questionnaires to assess health literacy around the topics of gestational weight gain [41] and pre-term birth [39]. One study [42] relied on proxy indicators of health literacy including reading fluency and comprehension. A detailed breakdown of health literacy measurement tools used is included in Table 3.

## Thematic summary of findings

### Theme one: Diverse definitions of health literacy

Most studies recognised health literacy to be a complex concept (n = 12), that encompasses personal attributes, skills, knowledge, and behaviours to manage health, apply information and navigate health systems. Four studies expanded this definition further to encompass situational determinants, social support, organisational factors and systems [31, 34, 36, 42]. In contrast, two studies did not provide an explicit definition of health literacy and refer to ‘health literacy’ as an assessment of knowledge [39, 41].

Variation in definitions of health literacy can create challenges when drawing inferences and conclusions from the evidence base. Variation is also seen across research methods used to measure and explore health literacy. For example, Kigawa et al. [41] utilise knowledge scores as a ‘proxy’ measurement of health literacy. Whereas other studies provided a detailed definition of health literacy as a multifactorial concept [30, 31] and focused their research on specific domains of health literacy. For example, ‘active engagement with care’.

### Theme two: Health literacy challenges can disproportionately affect minority ethnic women

All included studies provided evidence of women from ethnic minority backgrounds experiencing challenges across domains of health literacy including optimal access to maternity care, seeking and understanding health information, cultural and language barriers, comprehension and navigating complex care systems [32, 33, 35, 40, 42].

A number of studies provided evidence of minority ethnic women experiencing greater health literacy needs when compared with majority populations. The largest study, a secondary analysis of a US cohort study including 10,038 nulliparous women, found participants with lower health literacy were more likely to report being from an underrepresented racial or ethnic groups, among other characteristics including younger age, less educational attainment and medical insurance [36]. A large cross sectional survey study of 1,898 women across Denmark evidence disproportionate ethnic inequalities experienced by migrant women. After adjusting for age, parity, complications and occupation, lower levels of the health literacy domain ‘active engagement’ were reported among migrant European–0.15 (–0.26 to − 0.05), African − 0.19 (–0.40 to 0.02), Asian − 0.31 (–0.41 to − 0.21) and Syrian women − 0.42 (–0.58 to − 0.27) when compared to ethnic Danish women.

There is consistent evidence that health literacy challenges are most pronounced among minority ethnic women experiencing multiple forms of disadvantage [36, 43]. On an individual level, education, language and migration status appear to be important factors influencing health literacy, specifically the ability to seek information, communicate and self-advocate [31, 36, 41, 43]. From a systems perspective, the complexity of healthcare systems, quality of communication, and availability of culturally appropriate resources create multiple barriers to high quality maternity care [30, 32, 35, 40].

Explanation for additional barriers experienced by migrant women were explored in an explanatory mixed methods study of 501 postpartum women in Guangzhou urban villages [43]. Ethnicity, education, occupation, monthly income, household registration, delivery mode and social support were all significantly associated with maternal health literacy (*p* < 0.15) with minority ethnic women less likely to have adequate health literacy [43]. One participant said, “*I don’t have any relatives or friends here (Guangzhou) my elders are far away in the countryside*,* so there is little communication; I seldom communicate with my neighbours and mostly rely on myself or discuss with my husband or ask the doctor directly*” [43]. Further research with refugees and asylum seekers in Australia found that migrant women who spoke minority languages had lower health literacy scores with recent migration, experiences of trauma and unfamiliarity with the healthcare system creating multiple barriers to understanding and engaging with maternity care [34].

### Theme three: Poor health literacy can contribute to adverse outcomes and ethnic inequalities

Only two studies directly explored the impact of health literacy on health outcomes and inequalities. The largest, a multicentre US cohort study conducted by Yee et al. [36] identified associations between poor health literacy and adverse outcomes including: increased risk of caesarean birth (aRR, 1.11; 95% CI, 1.01–1.23), major perineal laceration (aRR, 1.44; 95% CI, 1.03–2.01), low birth weight babies (aRR, 1.33; 95% CI, 1.07–1.65) and babies having a 5-minute Apgar score less than 4 indicating compromise at birth (aRR, 2.78; 95% CI, 1.16–6.65) [36]. Observed increased risks of adverse outcomes were small, however minority ethnic women were disproportionately represented in this cohort of women with inadequate health literacy (72.2% Black, Hispanic, Asian women in inadequate health literacy group, compared with 31.6% Black, Hispanic, Asian women in adequate health literacy group) [36]. Notably when confounding variables in the analysis were accounted for the impact of ethnicity was not statistically significant, which may represent the impact of intersectionality of ethnicity and other social factors.

Comparatively, a Japanese cohort study examining the impact of health literacy on gestational weight gain found that lower health literacy scores did not translate into differences in the outcome of interest, appropriate gestational weight gain [41]. Though this finding may reflect limitations in the study’s health literacy measurement, which relied on knowledge scores rather than a comprehensive health literacy assessment tool [41]. Use of knowledge scores may not comprehensively measure health literacy as a multifaceted concept and present challenges when drawing conclusions about associated health outcomes.

Smaller studies found evidence of an association between adverse outcomes and lower health literacy among minority groups including poorer access to mental health services [38], lower attendance to care appointments [35] and lower eHealth literacy scores potentially limiting wider health literacy [37].

### Theme four: Limited evaluation of health literacy interventions

Several studies explored health literacy interventions aiming to address ethnic inequalities [30, 33, 34, 39, 41]. However, their effectiveness remains unclear due to limited evaluation. Two studies used knowledge-based questionnaires to assess health literacy which offer a proxy indicator of knowledge changes, pre- and post- intervention [39, 41]. However, this evaluation does not explore wider domains of health literacy. For example, personal skills, organisational factors and social resources. Two studies [33, 34] collected positive qualitative feedback on health literacy interventions but did not use validated health literacy measures or assess impact of interventions on clinical outcomes.

Robust evaluation is crucial to understanding complex inequalities and preventing unintended consequences that could worsen disparities. This was highlighted in research conducted on the Myanmar-Thailand border, which identified unintended consequences of a health literacy intervention. The study found that traditional health beliefs and ambiguous images led to widespread misunderstanding of health information posters, potentially worsening health literacy [42].

The most rigorous evaluation was provided by the MAMAACT cluster randomised controlled trial in Denmark, which tested a multi-component health literacy intervention designed to reduce ethnic disparities in stillbirth and infant health outcomes [30]. While the intervention improved some aspects of health literacy (recognising complications), it did not enhance women’s ability to navigate the healthcare system or improve clinical outcomes. This finding provided important insights into persistent organisational barriers that impact ethnic inequalities, even when personal health literacy is improved.

## Discussion

This scoping review aimed to map the extent and nature of research examining health literacy and ethnic inequalities in maternity care. The review identified that the extent of evidence examining health literacy and ethnic inequalities in maternity care is small, but growing, with a number of studies published in the last five years (*n* = 9). However, only 14 studies were eligible for inclusion in this review, and the quality of primary research is variable, particularly around testing the impact of interventions.

Nevertheless, this review provides further evidence of health literacy being a distinct and important determinant of maternal and neonatal health that contributes to disproportionate inequalities for minority ethnic women [20, 22, 23, 25]. There is no inherent reason for health literacy to differ by racial or ethnic identity. Rather, this review highlights how being from a minority ethnic group intersects with factors that can impact health literacy and exacerbate inequalities. Intersecting factors include migration status, language, support, comprehension and access to services. These findings are consistent with broader literature demonstrating the impact of intersectionality on health literacy and inequalities across various healthcare contexts [18, 20, 21].

A key finding in the review is variation in how the concept of ‘health literacy’ is defined and applied across different studies. Variation is also reflected in the assessment of health literacy, which further complicates the ability to compare findings across studies, populations and settings. Established instruments can support the exploration of health literacy as a multidimensional concept but have largely been developed and tested in Western, English-speaking populations which may not apply to the exploration of ethnic inequalities. Other tools can oversimplify the concept of health literacy measuring knowledge scores, reading ability or comprehension, though such tools may be more easily applied across diverse populations.

The current evidence base does not yet establish which health literacy measurement tools are the most appropriate or robust for researching health literacy in the context of ethnic inequalities. Notwithstanding, detailed exploration of health literacy is valuable in adding context to findings that are not solely based on race and ethnic identification but explore wider intersecting factors [22, 25]. This is particularly important when considering inequalities that can be masked within and between aggregated minority ethnic groups, across settings.

The main methodological limitations to the existing evidence base are small sample sizes, a lack of appropriate comparison groups, inadequate measurement of health literacy, and limited follow up of clinical outcome measures. Quantitative evidence is largely limited to small descriptive studies which does not provide understanding of causal relationships between health literacy and ethnic inequalities. Only one study used a randomised controlled trial design to evaluate an intervention [30], making it challenging to draw conclusions around the impact of health literacy interventions on inequalities. Low quality studies risk exacerbating inequalities through the use of poorly designed or culturally inappropriate interventions [44].

There is a paucity of detailed qualitative research undertaken with women from minority ethnic backgrounds which could develop knowledge around the mechanism in which health literacy influences ethnic inequalities in maternity care. Only one qualitative study undertook detailed exploration of women’s experiences, with other qualitative studies exploring the perspectives of healthcare professionals. Though currently limited (*n* = 3), mixed methods research appears to have merit in better understanding health literacy holistically within the context of ethnic inequalities.

The largest geographical gaps in the research are across Africa, South America, the Middle East and Eastern Europe. It was notable to the authors of this paper (based in the United Kingdom) that there were no primary research studies exploring the intersection of health literacy and ethnic inequalities in the United Kingdom. This is despite calls to address ethnic inequalities in UK maternity care [45] and national reports highlighting Black, Asian, and minority ethnic women feeling unsupported to make informed decisions, not being listened to and facing difficulties accessing care appropriately [9, 12–15]. Given the intergenerational effects of maternal health outcomes, addressing health literacy barriers in maternity care could have benefits extending beyond immediate maternal health, to child and family wellbeing. Critically, exploring ethnic inequalities through a health literacy lens offers a novel perspective and aligns with issues highlighted in UK national reports and qualitative studies.

## Strengths and limitations

The review used a comprehensive search strategy and adhered to PRISMA-ScR guidelines promoting transparency [27]. Studies were excluded if they did not directly examine ‘health literacy’ as a defined concept. The authors recognise this is a limitation of the review, however as the primary purpose of the review was to scope existing literature around the topic of ‘health literacy’, this term was used as a screening tool to examine the extent of research within this defined topic area. Future reviews may consider widening the search to include terms that describe elements of health literacy. For example, communication, education, navigation of health systems. A realist approach could be considered to explore variation across definitions of health literacy within the context of ethnic inequalities.

The screening of papers was conducted by the primary author and whilst quality assessment was considered, a formal categorisation was not undertaken. This is consistent with scoping review methodology but does limit the scope of this review to a mapping exercise rather than evaluating and synthesising the evidence base. Thus, the interpretation of findings should be treated with greater caution than would be the case in a formal evidence synthesis following a systematic review. The review contains a small number of studies, with diverse research design and health literacy assessment tools, which limits the ability to draw generalisable conclusions. The wide geographical spread of existing research, combined with its focus on ethnic inequalities affecting minority groups in different settings, further complicates the generalisability of the findings to specific populations or contexts. Though these limitations restrict the strength of the conclusions, they also emphasise the importance of future context specific research in this area.

## Conclusion

Health literacy is an important but understudied determinant of ethnic inequalities in maternity care. Health literacy challenges can disproportionately impact minority ethnic women and appear to be compounded by intersecting factors such as education, language barriers and migration status. Optimisation of health literacy may serve to reduce persistent global ethnic inequalities in maternity care outcomes, however evidence-based development of interventions aiming to tackle inequalities is limited by a paucity of studies leading to large research gaps in this area.

Consequently, there is a need for high-quality in-depth research to explore health literacy and ethnic inequalities in maternity care using defined terms and measurement tools validated for use across diverse linguistic and cultural settings. Developed understanding of women’s health literacy profiles, and the means to which health literacy contributes to ethnic inequalities, can support development of targeted, equity focused interventions. Robust evaluation of potential interventions is integral to reducing inequalities and preventing inadvertent exacerbation of inequalities.

## Data Availability

Data sharing is not applicable to this article as no datasets were generated or analysed during the current study.
